# Dataset for time-lapse ultrasonic tomography of a granite slab under uniaxial compression test

**DOI:** 10.1016/j.dib.2018.08.151

**Published:** 2018-08-30

**Authors:** Qi Zhao, Tai-Ming He, Johnson Ha, Kaiwen Xia, Giovanni Grasselli

**Affiliations:** a Department of Civil Engineering, University of Toronto, ON M5S 1A4, Canada; b Key Laboratory of Seismic Observation and Geophysical Imaging, Institute of Geophysics, China Earthquake Administration, Beijing 100081, China

## Abstract

This data article includes raw data for time-lapse ultrasonic tomography measurements during a uniaxial compression test. Two sets of experimental data are included: first, the ultrasonic tomography (UT) observation (i.e., waveform) data at each 20 MPa axial stress step during the uniaxial loading test; and second, the stress-strain curve of the uniaxial compression test. A numerical model based on the combined finite-discrete element method (FDEM) was used to improve the understanding of the experimental results. The model file and the simulated acoustic emission (AE) data extracted from the simulation results are also included in this article. Data sets presented in this article help to improve understanding of the progressive rock failure process at microscopic and macroscopic scales.

**Specifications Table**

**Table:** 

Subject area	Rock mechanics, Rock engineering, Geophysics
More specific subject area	Rockburst, Ultrasonic tomography (UT), Acoustic emission (AE)
Type of data	Text file, MATLAB data (.mat).
How data was acquired	Waveform data was acquired with an ultrasonic sensor array consisting of 26 transducers surrounding a granite slab that was tested by uniaxial compression test. Numerical model was created and run by the software package Irazu 2.0.0 (Geomechanica Inc.). Simulated AE data was extracted using ParaView and saved in MATLAB.
Data format	Raw data
Experimental factors	Fangshan granite slab 110 mm wide, 220 mm long, and 30 mm thick
Experimental features	26 transducers surrounding the sample conducted a time-lapse UT at each 20 MPa stress interval during a uniaxial compression test
Data source location	Laboratory test was conducted at the Key Laboratory of Seismic Observation and Geophysical Imaging, Institute of Geophysics, China Earthquake Administration, Beijing, China
	The numerical simulation was conducted at the Department of Civil Engineering, University of Toronto, Canada.
Data accessibility	Data are presented in this article
Related research article	T.-M. He, Q. Zhao, K. Xia and G. Grasselli. (2018). Understanding progressive rock failure and associated seismicity using ultrasonic tomography and numerical simulation, *Tunnelling and Underground Space Technology*, 81, 26–34.

**Value of the data**

• This is the first set of data that combines UT and FDEM numerical simulation to investigate progressive rock failure
• The time-lapse UT data acquired during the uniaxial loading test depicts the gradual rock deformation and failure processes
• The calibrated numerical model parameters can be used to simulate granite behavior
• Simulated AE events can be used to compare to other laboratory experiments and numerical simulations

## 1 Data

The data described in this paper provides information on time-lapse UT measurements acquired during a uniaxial compression [1]. Seven stages of UT observation data are included, which were acquired at the 0 MPa (0 kN) and every 20 MPa (66 kN) incremental axial loading step. At each stage, all the waveforms between source–receiver pairs are provided. Moreover, the stress–strain curve of the uniaxial compression test is provided. An FDEM model that is calibrated based on the sample properties and test is also provided. The simulated AE data extracted from the simulation results are provided as well.

The dataset presented here is the first time the time-lapse UT and FDEM numerical simulation are combined to investigate progressive rock failure. This data article allows the work in He et al. [1] to be reproduced and compared with other laboratory experiments and numerical simulation data.

## 2 Experimental design, materials and methods

### 2.1 Uniaxial compression test and ultrasonic tomography

The rock sample investigated in this study is a Fangshan granite slab that is 110 mm in width, 220 mm in height, and 30 mm in thickness. The uniaxial compression test was conducted on this sample using an MTS–1000 kN hydraulic test system, and the axial load was increased at a constant rate of 6 kN/min, until the failure of the sample.

There are 26 ultrasonic transducers surrounding the sample [1]. At each stage of the UT observation, one side of the transducer array sent out ultrasonic wave signals (1 MHz pulse) and transducers on the other three sides acted as receivers. This procedure was carried out following counterclockwise direction.

### 2.2 Numerical simulation

The FDEM model was built based on the cross-section of the laboratory setup [1]. It consisted of a 220 mm × 110 mm longitudinal section representing the rock sample and two rectangles representing the loading platens. The model had an average mesh element size similar to the rock sample averaged grain size of 2.6 mm. The model recreated the mineral heterogeneity of the Fangshan granite by randomly assigning mineral properties to the model elements according to the relative percentage of mineral components in the actual sample, namely, 67% feldspar, 23% quartz and 10% biotite.
